# Green Communication for Wireless Body Area Networks: Energy Aware Link Efficient Routing Approach

**DOI:** 10.3390/s18103237

**Published:** 2018-09-26

**Authors:** Muhammad Anwar, Abdul Hanan Abdullah, Ayman Altameem, Kashif Naseer Qureshi, Farhan Masud, Muhammad Faheem, Yue Cao, Rupak Kharel

**Affiliations:** 1 School of Computing, Universiti Teknologi Malaysia, Johor Bahru 81300, Malaysia; anwar.muhammad@ieee.org (M.A.); hanan@utm.my (A.H.A.); farhan.contact@gmail.com (F.M.); muhammad.faheem@agu.edu.tr (M.F.); 2 College of Applied Studies and Community Services, King Saud University, Riyadh 11564, Saudi Arabia; aaltameem@ksu.edu.sa; 3 Department of Computer Science, Bahria University Islamabad, Islamabad 44000, Pakistan; knaseer.buic@bahria.edu.pk; 4 Faculty of Life Sciences Business Management, University of Veterinary and Animal Sciences, Lahore 54000, Pakistan; 5 Department of Computer Engineering, Abdulla Gul University, Keyseri 38000, Turkey; 6 Department of Computer and Information Sciences, Northumbria University, Newcastle upon Tyne NE2 1HL, UK; 7 School of Engineering, Manchester Metropolitan University, Manchester M15GD, UK; r.kharel@mmu.ac.uk

**Keywords:** wireless body area networks (WBANs), wearable sensors, routing protocol, energy efficiency

## Abstract

Recent technological advancement in wireless communication has led to the invention of wireless body area networks (WBANs), a cutting-edge technology in healthcare applications. WBANs interconnect with intelligent and miniaturized biomedical sensor nodes placed on human body to an unattended monitoring of physiological parameters of the patient. These sensors are equipped with limited resources in terms of computation, storage, and battery power. The data communication in WBANs is a resource hungry process, especially in terms of energy. One of the most significant challenges in this network is to design energy efficient next-hop node selection framework. Therefore, this paper presents a green communication framework focusing on an energy aware link efficient routing approach for WBANs (ELR-W). Firstly, a link efficiency-oriented network model is presented considering beaconing information and network initialization process. Secondly, a path cost calculation model is derived focusing on energy aware link efficiency. A complete operational framework ELR-W is developed considering energy aware next-hop link selection by utilizing the network and path cost model. The comparative performance evaluation attests the energy-oriented benefit of the proposed framework as compared to the state-of-the-art techniques. It reveals a significant enhancement in body area networking in terms of various energy-oriented metrics under medical environments.

## 1. Introduction

The technological advancement has brought a revolution in today’s human life. It has changed the way of human’s working in every field of life such as home automation, smart cities, environment monitoring, and prediction [1,2,3,4,5]. Despite all these advancements, humans still face many challenges. The current forefront challenge in healthcare is fast growing of world population and decreasing number of healthcare facilities in proportion to the population ratio. According to the US Census Bureau, it is predicted that the population of aged people in the world will be doubled up to 761 million in 2025 from 375 million in 1990 [6]. Generally, the elderly suffer from various chronic diseases, thus they require continuous medical care. Most of them have to stay in hospitals or remain under constant supervision of a medical professionals, otherwise their lives may be at risk. Every year, thousands of people die due to fatal or chronic diseases. The most common reason for such fatal diseases is lack of timely diagnoses. Research has revealed that most of these diseases may be controlled if identified at their initial stages [7]. Therefore, there is a pressing need to develop proactive and affordable healthcare systems for continuous health monitoring without any attendants and to diagnose the diseases at their early stages.

In order to address the healthcare challenges, researchers from academics and medical sciences have introduced wireless body area networks (WBANs). This is a promising technology in healthcare which consists of smart biomedical sensor nodes (BSNs) that can be implanted or worn on human body. The BSNs are equipped with limited computational resources including sensing and collecting data from human body and sending it to medical center for further processing [8,9]. WBAN is an economical healthcare system for medical professionals and patients. It gives the advantage of mobility to patients, allowing them to be engaged in their routine activities instead of staying in hospital or under constant supervision of a medical professional [10].

**Figure 1:**
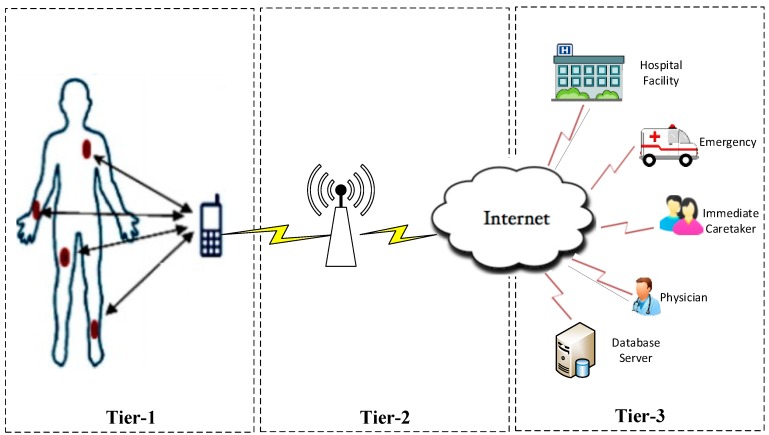
Architecture of WBAN communications.

WBANs emerged from wireless sensor networks (WSNs) [11]. However, they are somehow diverse due to some intrinsic challenges. WBAN three tiers communication architecture is shown in Figure 1. Tier-1 (Intra-WBAN) refers to communications among BSNs and body node coordinator (BNC) where nodes send their sensory data to BNC. Tier-2 (inter-WBAN) denotes the communication of BNC with remote medical site. Tier-3 (Beyond-WBAN) consists of medical servers for real-time diagnosis, history of patients record keeping and generating alert to the emergency services, medical professionals, and immediate caretakers of the patients [12].

In intra-WBAN communication, reliable data transmission is a critical challenge due to dynamic and impulsive behavior of BSNs [13]. Sensor nodes have short battery life, the optimal energy consumption is the major problem in WBANs [14,15]. If a sensor node runs out of battery and is unable to transmit physiological signals, it will be life threatening to the patient. Hence, the sensor nodes should survive longer.

Almost 80% of the sensor energy in WBANs is utilized by communication processes [16,17]. The network lifetime of BSNs can be enhanced by optimizing the communication process. Due to the resource limitations and short communication range of BSNs, direct communication between BSNs and BNC is not suitable because of path loss issues [18,19]. Direct communication consumes more energy. Therefore, multi-hop communication is comparatively more appropriate for WBANs because it balances out the energy more efficiently [20]. BSNs in multi-hop communication, in which sensor nodes send data to their neighboring nodes instead of sending directly to the BNC [21,22]. In multi-hop communication, the selection of next-hop as a forwarder node is the most critical part of routing protocols. The existing routing protocols in WBANs present several tradeoffs for selecting the next-hop. However, these protocols attempt to choose the route with shorter path instead of route with best quality path. Hence, these protocols lead to high power consumption in WBANs. Towards this end, this paper presents a green computing framework focusing on an energy aware link efficient routing approach for WBANs (ELR-W). Here, it is noteworthy that literature did not consider multipath oriented path loss-oriented impacts while calculating link efficiency. However, our major novelty is on incorporating multipath path loss-oriented packet reception rate, and interference effect on link quality calculation along with distance and residual energy considerations. Our overall contribution in this paper can be summarized as follows:

- Firstly, a link efficiency-oriented network model is presented considering beaconing information and network initialization process.
- Secondly, a path cost calculation model is derived focusing on energy aware link efficiency.
- A complete operational framework ELR-W is developed considering energy aware next-hop link selection by utilizing the network and path cost model.
- The comparative performance evaluation has been carried out focusing on energy-oriented metrics under WBANs medical environments.

Furthermore, the related previous work is presented in Section 2 of this paper, modeling detail of the proposed ELR-W framework is discussed in Section 3. Section 4 discusses simulation results and analysis, followed by Section 5 where the conclusion of this study and future direction are presented.

## 2. Related Work

The BSNs in WBANs are heterogeneous in nature and have very limited resources. The effectiveness of routing protocols for energy efficient route selection depends on the optimal utilization of the resources. Javaid et al., in [23], proposed a mobility supporting adaptive threshold based thermal aware energy efficient multi-hop protocol (M-ATTEMPT) for WBANs. They employed heterogeneous BSNs on human body. The protocol used direct communication for sensitive and on demand data traffic whereas multi-hop communication for ordinary data traffic. For multi-hop communication, this protocol selects forwarder node based on less hop-count to the BNC, and high available energy of the neighboring node. M-ATTEMPT addresses the challenges of heat generated by implanted sensor nodes and mobility issues in WBANs. However, when a node’s temperature goes across the threshold level after receiving a data packet, it retransmits that packet recurrently, which causes more consumption of energy and has low network reliability [24]. 

Maskooki et al., in [25], introduced an opportunistic routing for WBAN. They stated that the postural movement of body can decrease the performance of a WBAN. Therefore, the mobility is a big challenge for reliable data delivery. To overcome this issue, they proposed an opportunistic routing. They presented an idea of using relay node at right place on body so that most of the communication can be taken place directly though relay node. They placed a sink node on the wrist and a BSN on the chest. When walking, the patient’s hand would move forward and backward, the BSN would directly send data to the sink when the wrist was on front side. On the other hand, the BSN uses a relay node to transmit the data when the wrist is behind the body. In this way, the BSNs have an opportunity to directly transmit the data at line of sight (LOS) for a longer time. However, this protocol is unable to select the routing path when a BSN is at the same distance from the sink and relay node. Moreover, deployment of a relay node requires additional network cost [26].

Liang et al., in [27], stated that the quality of wireless link in WBANs varies frequently due to body shadowing which results in low reliability and energy deficiency. They proposed an energy efficient routing scheme (EERS) based on tree structure. This scheme selects an energy-efficient routing path and adaptively sets transmission power for BSNs. Simulation results of EERS present the improvements in terms of mean delay, energy consumption, and packet reception ratio (PRR). However, this protocol faces overhead in adaptive transmission power [24]. Moreover, Ahmed at al., in [28] proposed a cooperative link-aware and energy efficient protocol (Co-LAEEBA) aiming for energy efficient routing in WBANs. They proposed a cost function based on distance and residual energy level to select the best feasible route towards the sink node. This protocol shows better performance in terms of energy efficiency. However, it results in high packet drop [29].

In stable increased-throughput multi-hop protocol for link efficiency (SIMPLE) [30], the authors placed eight fixed BSNs on human body. They placed two BSN close to the BNC for monitoring the level of glucose and ECG. These BSNs originate sensitive data which needs a high level of reliability and network lifetime. These BSNs use direct communication to sink node to forward their data, whereas other sensor nodes follow multi-hop communication and send their data to their parent or forwarder nodes. In this protocol, the nodes generating critical data are placed near to the BNC which are mostly selected as forwarder nodes and act as relay node for others. Due to this, these nodes deplete their energy rapidly which results into failure of sending the critical information at first. The same forwarder selection criterion is used in iM-SIMPLE [25] which curtails the overall network reliability in WBANs [18].

Sahndhu et al., proposed BEC [31] targets to balance out the energy utilization in WBANs. The protocol follows multi-hop topology to send data from farthest node to BNC. Relay nodes are elected at the initial stage on the basis of cost function proposed by the authors. All other BSNs send their data to their designated relay nodes using time division multiple access (TDMA). The nodes with less energy than the threshold value forward critical data only. The protocol promotes the packet delivery and decreases the packet loss in the network. However, the selected relay nodes expend their energy very fast which decreases the overall network lifetime [32]. Adhikary et al., in [33], proposed a routing protocol aiming to optimize energy consumption in WBANs. In this protocol, the authors placed additional fixed nodes to act as forwarders for other BNSs. They proposed route selection criteria based on transmission power and energy of intermediate BSNs, velocity vector of the receiving node, and distance from the BNC. The protocol performs well in terms of network lifetime. However, the strategy of use of additional relay nodes is uncomfortable for the patients.

Ha [34] introduced even energy-consumption and backside routing (EEBR) for WBANs. In this work, the authors placed BSNs on both front and backside of the patient body. This protocol addressed the issues pointed out in M-ATTEMPT routing protocol and provided communication coverage at the backside of the body. A cost function based on residual energy and number of hop-counts is proposed to select the route. The path with minimum standard deviation of cost function is selected for data delivery. However, the nodes placed on backside of the body experience high path loss because of not considering link efficiency for route selection. Ayatollahitafti et al., proposed a next-hop selection algorithm [35] for WBANs. To balance the energy consumption, multi-hop communication strategy is exploited based on hop-counts and cost function. This algorithm performed well against the benchmark protocol. However, the use of buffer size in its cost function, for selection of next-hop causes delay in data transmission. Ullah et al. proposed a dual sink clustering approach in BAN (DSCB) [36] which uses two sinks. Each sink maintains its own cluster to avoid contention in the network. The BSNs send data to their designated sink only. The route is chosen on the basis of the cost function which is composed of energy, distance, and transmission power. Signal to noise ratio (SNR) is used to compute the required transmission power by BSNs. The protocol uses the resources more effectively and improves the network lifetime. However, deployment of dual sink requires additional network cost [26].

## 3. Green Computing for WBANs

ELR-W protocol aims to dynamically select the best next-hop from each BSN to the BNC based on residual energy, link efficiency, number of hop counts, and distance to the BNC. ELR-W is a multi-hop routing protocol in which each BSN generates data packets and sends it to the neighboring node. The receiving node then forwards these packets to BNC. The best next-hop selection is the main idea in ELR-W. In general, selecting a path with a lower number of hop counts to BNC is an effective approach. However, a path with a greater number of hop counts that uses more energy of intermediate nodes may be considered better for the sake of energy balancing in the network. In this situation, the proposed protocol increases the path cost having nodes with lower energy level. This means that a neighboring node having greater residual energy will be selected over a neighboring node with less residual energy. The distance between nodes and signal strength is also a significant parameter for next-hop selection which directly affects energy consumption. This approach balances the energy utilization among all BSNs which results in more stable and improved network lifetime of WBANs.

### 3.1. Link Efficiency Oriented Network Model

**Figure 2:**
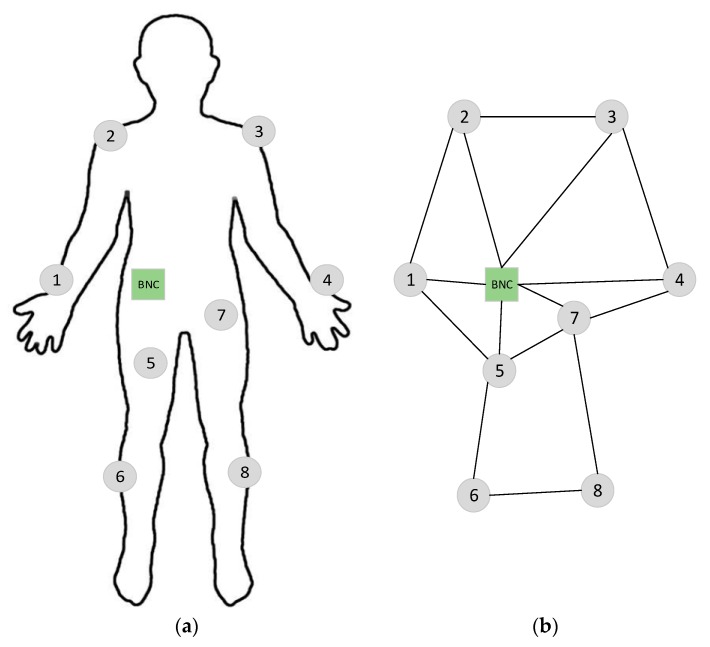
Network topology (**a**) Physical Topology (**b**) Logical topology.

The physical and logical topology of WBANs network model with eight BSNs and one BNC is exhibited in Figure 2. These BSNs generate heterogeneous types of data and send it to the BNC located on the body waist. The BNC simply receives data from the BSNs and does not generate any data at its own. Each sensor node determines its neighboring nodes according to its communication range. The logical topology produced from Figure 2a is shown in Figure 2b. In the logical topology, the nodes denote the sensor nodes, whereas edges indicate the wireless connections between these sensor nodes. The wireless connections are shown according to the communication range of the sensor nodes.

ELR-W is developed with the following assumptions:

- All BSNs are fixed on a human body as exhibited in Figure 2a and no node is implanted.
- Each node possesses the same energy, processing power, and interfaces.
- All BSNs have fixed and limited transmission power.
- Human body movement is not considered in this research.

Considering the shorter distance does not always lead to lower path loss values. Due to the multipath oriented interference effect, shorter distance might lead to higher path loss values. Here it is highlighted that to incorporate the similar situations. The link quality estimation is considered as another parameter for helping in selecting the next-hop in case of shorter distance and dense environments.

#### 3.1.1. Hello Packets (HP)

Hello packets are used to maintain adjacencies between neighboring nodes. BSNs share their updated residual energy, number of hop-counts, link efficiency, and distance to the BNC by circulating the HPs periodically. The field of the HP header is elaborated in Table 1.

The link efficiency can be calculated based on the receive signal strength indicator (RSSI), the link quality indicator (LQI), and packet reception rate (PRR) [37]. However, this work determines the link efficiency based on PRR because it is a memory efficient method and requires little computations. The link efficiency can be computed in Equation (1).

(1) $$\;LE=\sum \frac{PR_{N}}{PS_{S}}\;$$

where *LE* denotes link efficiency, $PR_{N}$ refers to the number of packets received at the neighbor node, and $PS_{S}$ indicates the number of packet sent from the source node. 

The proposed protocol keeps track of residual energy of each BSN by calculating the consumed energy in each round using Equation (2).

(2) $$\;RE=E_{init}-E_{cons}\;$$

where *RE* is the residual energy of a BSN, $E_{init}$ is the initial energy, and $E_{cons}$ is the energy consumed in each round.

The distance from the source node to BNC can be calculated from *X* and *Y* coordinates as in Equation (3).

(3) $$d_{\left(i,BNC\right)}\;=\;\sqrt{{\left(X_{i\;}-X_{BNC}\right)}^{2}+\;{\left(Y_{i\;}-Y_{BNC}\right)}^{2}}\;$$

#### 3.1.2. Neighbor Table (NT)

Each sensor node stores status information of its adjacent neighboring nodes. This information is collected from HPs received from each neighbor node. Each time a BSN receives HP from its neighbor node, it updates its information in NT. The procedure for constructing and updating NT is demonstrated in Algorithm 1.

**Algorithm 1**: Neighbor table construction algorithm of ELR-W protocol at node *i***Notations:** HP = Hello packet $RE_{j}$ = Residual energy of neighbor node *j* $LE_{i,J}$ = Link efficiency between node *i and* node *j* $HC_{j,BNC}$ = Number of hop-counts from neighbor node *j* to ***BNC*** $d_{i,j}$ = Distance between nodes *i* and *j* (NT) = Information in neighbor table (HP) = Information in Hello packets **Input:** *HPs* from a neighboring node *j* **Process:** *1.* ***start*** *2.* ***for***
*each HP*
***do*** 3. ***if***
$HP\left(RE_{J},\;LE_{i,J},\;HC_{i,BNC},\;d_{i,j}\right)\neq \mathrm{NT}\left(RE_{J},\;LE_{i,J},\;HC_{i,BNC},\;d_{i,j}\right)$
***then*** 4. update record for neighbor information in neighbor table 5. $RE_{j}\left(NT\right)\;\leftarrow \;RE_{j}\left(HP\right)$ 6. $LE_{i,j}\left(NT\right)\;\leftarrow \;LE_{i,j}\left(HP\right)$ 7. $d_{i,j}\left(NT\right)\;\leftarrow \;d_{i,j}\left(HP\right)$ 8. $HC_{i,BNC}\left(NT\right)\;\leftarrow \;HC_{i,BNC}\left(HP\right)$ 9. ***else*** *10.* *Discard HP* *11.* ***if***
$HP\left(E_{\left(Res\right)J},\;LE_{i,J},\;HC_{i,BNC},\;d_{i,j}\right)$
*= null*
***then*** 12. add record in neighbor table 13. $RE_{j}\left(NT\right)\;\leftarrow \;RE_{j}\left(HP\right)$ 14. $LE_{i,j}\left(NT\right)\;\leftarrow \;LE_{i,j}\left(HP\right)$ 15. $d_{i,j}\left(NT\right)\;\leftarrow \;d_{i,j}\left(HP\right)$ 16. $HC_{i,BNC}\left(NT\right)\;\leftarrow \;HC_{i,BNC}\left(HP\right)$ 17. ***else*** go to line 3 *18.* ***end if*** *19.* ***end if*** *20.* ***end for*** *21.* ***end***

### 3.2. Path Cost Estimation

According to the Dijkstra algorithm [38], selecting the path with a lower number of hop-counts to the BNC is an effective approach. However, the path with a greater number of hop counts using the higher energy of intermediate nodes may be considered better for balancing energy consumption among the nodes. Link efficiency (LE) between the nodes directly affects the energy consumption. The route with low link efficiency may lead to packet loss and retransmission attempts which consume high energy. The existing routing protocols always attempt to choose the shortest path based on the distance to the BNC. However, unlike other routing protocols, this work considers link efficiency as well as shorter path for selecting the next-hop for data transmission. This framework introduces a novel path cost function (*PCF*) based on residual energy (*RE*), link efficiency (*LE*), hop-counts (*HC*), and distance (*d*) to the BNC. The BSN with the least value of the PCF is chosen as the next-hop for packet forwarding. The value of path cost function is calculated in Equation (4).

(4) $$\;PCF=\underset{\forall N_{i}\in N}{\sum }\left[\alpha \times \frac{1}{RE}+\beta \times \frac{1}{LE}+\gamma \times HC+\delta \times d\right]\;$$

where α, β, γ, and δ denote the weighting factors for the residual energy (RE), link efficiency (*LE*), number of hop counts (*HC*), and distance to the BNC (*d*) respectively. Each weighting factor is assigned a value according to its priority so that α+β+γ+δ=1. In order to assign the priority to each parameter in the next-hop selection, the weighting factors are assigned the following values.

 α=0.4 

 β=0.3  γ=0.2 

 δ=0.1 

#### 3.2.1. Routing Table (RT)

When nodes receive the hello packets from their neighboring nodes, they update their NT which is used to update the RT. If a packet is received for the first time from a sender node, a new entry is created in the RT. The RT contains ‘neighbor IDs’ and ‘path cost’ values of each neighbor node. The next-hop is selected based on the least value of the PCF.

#### 3.2.2. Radio Energy Model

The ELR-W protocol uses the basic model for radio energy consumption discussed in [39]. In this model, energy consumption to transmit and receive *k* number of bits over distance *d* is determined according to the following equations.

(5) $$\;E_{Tx}\;\left(k,d,n\right)=\;E_{Tx-elect}k+E_{amp}\left(n\right)kd\;$$

(6) $$\;E_{Rx}\left(k\right)=E_{Rx-elect}\;k\;$$

where $E_{Tx}$ is the energy utilization for transmitting and $E_{Rx}$ is for receiving the data packet. While $E_{Txelect}$ and $E_{Rxelect}$ indicate the energy consumption by the radio operations for the purpose of transmission and reception correspondingly. $E_{amp}$ is the energy utilization for amplification and n is the coefficient used for path loss. The values of these parameters depend on the hardware transceivers. We consider these parameters for Nordic nRF2401 [39] which is a low power single chip transceiver commonly used for body area networks. The parameter values are presented in Table 1.

#### 3.2.3. Path Loss Model

The propagation of wireless signals in WBANs experience shadowing and fading effects of the human body. Several more complex path-loss prediction models are available in the literature such as [40,41,42,43]. These models have been for different environment specific variations and have their own pros and cons. However, we exploit a Friis formula-based path loss model as used in our benchmark protocols and by other recent studies in WBANs [18,44,45]. The usage of more complex path-loss prediction models requires more computation in signal characterization leading to higher energy consumption. Considering our energy centric communication model development for wireless body area networking, we employ the simplistic path loss models. This model defines PL as a linear function of the distance *d* between the nodes. The path loss $PL_{ij}$ in decibel (dB) between node *i* and node *j* can be formulated in Equation (6).

(7) $$\;PL_{i,j}\left(d\right)=PL_{0}+10\left(n\right)\log_{10}\frac{d_{i,j}}{d_{0}}+X_{\sigma }\;$$

where $PL_{0}$ is the path loss at a reference distance $d_{0}$ which is considered 10 cm in our simulation similar to [26], *n* is the path loss coefficient which is considered 2 as it is implemented in free space, X represents Gaussian random variable [45], and σ is the standard deviation [46]. The $PL_{0}$ can be further derived in Equation (7).

(8) $$\;PL_{0}=10\log_{10}\frac{{\left(4\pi d_{0}\right)}^{2}}{s\lambda^{2}}\;$$

where  s denotes the speed of light and λ represents the wavelength.

### 3.3. ELR-W: Operational Steps 

**Figure 3:**
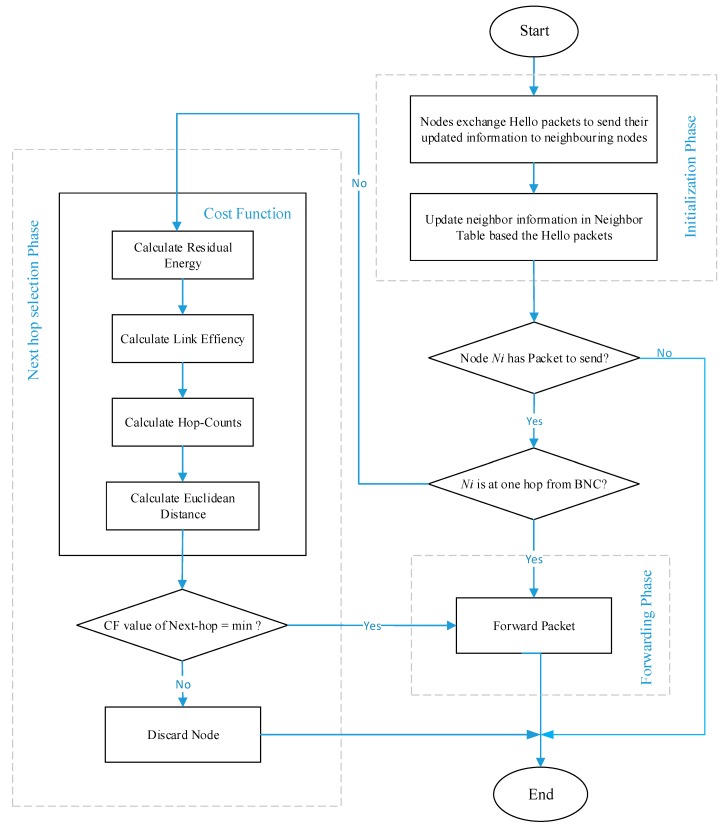
Flow chart of ELR-W protocol.

The proposed protocol has three phases; initial phase, next-hop selection, and forwarding phase. The flow chart of ELR-W is shown in Figure 3.

#### 3.3.1. Initialization Phase

In this phase of ELR-W protocol, BNC broadcasts a hello packet (HP) to convey its status and position on the body. All BSNs receive and store the position information of the BNC. Then each BSN broadcasts HP containing node ID, its energy status, location information, number of hops, and distance to the BNC. Thus, all BSNs update their neighbor’s information in their NT.

#### 3.3.2. Next-Hop Selection Phase

For the purpose of improving routing efficiency of a protocol, next-hop selection criterion is most important. In this phase, the proposed ELR-W protocol selects best available next-hop for the packet forwarding. The ELR-W makes this decision based on the path cost stated in Equation (4). The node with least value of path cost is preferred as the next-hop. The algorithm for next-hop selection is presented in Algorithm 2.

#### 3.3.3. Forwarding Phase

Once the next-hop is elected, the BSN will send data packet to the selected node which will further transfer packet to BNC. BNC is a gateway for all BSNs, which receives data from BSNs and transmits to medical server though internet.

**Algorithm 2:** Next-hop selection procedure**Notations:** $N_{i}$ = Source node $NH_{i}$ = Next-Hop node for $N_{i}$ *BNC* = Body Node Coordinator *NT* = Neighbor Table *PCF* = Path Cost Function **Input:** records in *NT* **Process:** *1.* ***start*** 2. ***if***
$N_{i}\;$ is at one hop to BNC ***then*** 3. send packet directly to BNC *4.* ***else*** 5. ***for*** each record in *NT*
***do*** 6. Calculate $PCF=\underset{\;}{\sum }\left[\alpha \times \frac{1}{E_{Res}}+\beta \times \frac{1}{LE}+\gamma \times HC+\delta \times d\right]$ 7. List *RT*
← PCF value of each neighbor node in *NT* 8. $NH_{i}\;$← min(*RT*) 9. ***end for*** 10. ***end if*** 11. ***end***

## 4. Experimental Results 

The experiments are performed by considering eight BSNs and one BNC are placed on human body as shown in Figure 2. All BSNs generate constant bit rate (CBR) traffic. We considered simulation parameters for Nordic nRF2401 [47] which is low power single chip transceiver commonly used for body sensor networks. The parameter values are presented in Table 2. A number of experiments have been performed using NS-2 to assess the performance evaluation of proposed ELR-W protocol. The results are compared with M-ATTEMP [22] and iM-SIMPLE [25] protocols. M-ATTEMP and iM-SIMPLE are selected because of their close relevancy to the proposed protocol. We have modified our implementation considering literature’s parameter consideration and way of calculation for reflecting comparative analysis. The performance of ELR-W protocol is measured based on throughput, residual energy, and packet loss. 

**Figure 4:**
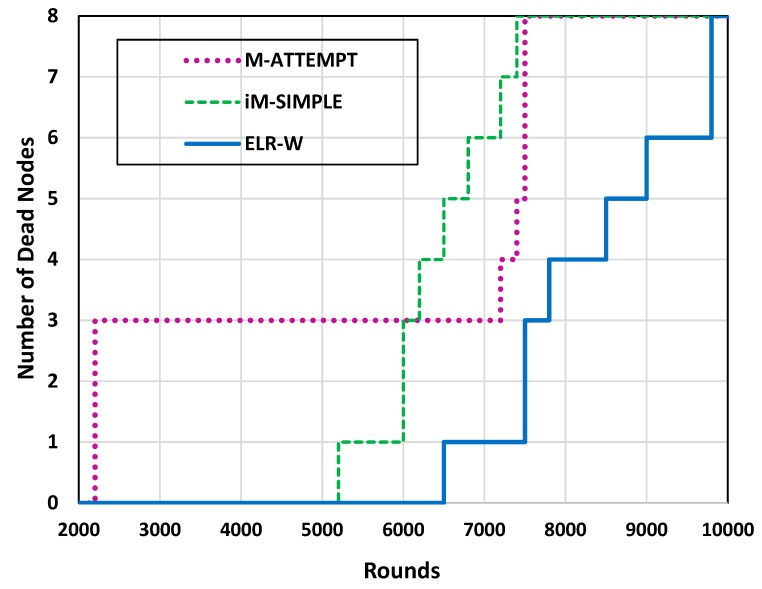
Analysis of network lifetime.

**Figure 5:**
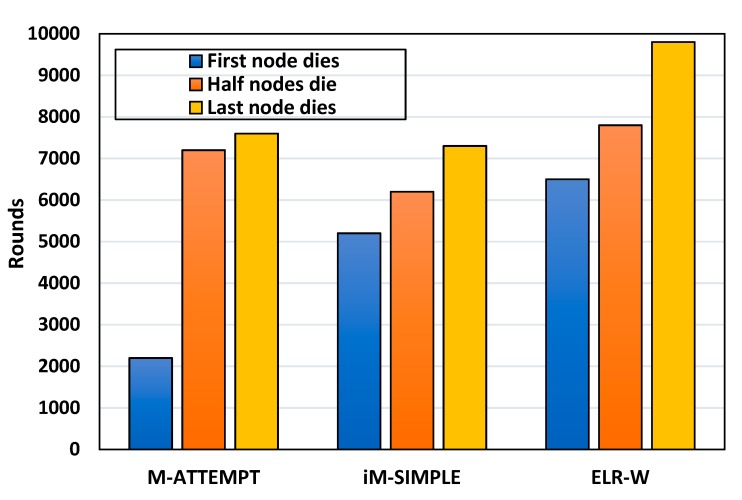
Analysis of network lifetime.

In WBANs, the network lifetime depends upon the life of BSNs. The network lifetime of ELR-W, M-ATTEMPT, and iM-SIMPLE can be viewed in Figure 4 and Figure 5, which demonstrate the comparison of proposed ELR-W with M-ATTEMPT and iM-SIMPLE in terms of dead nodes. The analysis depicts that in M-ATTEMPT the first three nodes died at 2200 rounds due to heavy load generated on these nodes. In iM-SIMPLE and ELR-W, the first node dies at 5200 and 6500 rounds correspondingly. Figure 5 reveals that the entire nodes of M-ATTEMPT and iM-SIMPLE die at 7500 and 7300 rounds respectively, while ELR-W protocol is able to live up to 9800 rounds. Hence, it shows that the ELR-W protocol has greater network lifetime in contrast to M-ATTEMPT and iM-SIMPLE. Moreover, the statistical analysis indicates the network lifetime of ELR-W is 30% and 34% longer than M-ATTEMPT and iM-SIMPLE, respectively.

**Figure 6:**
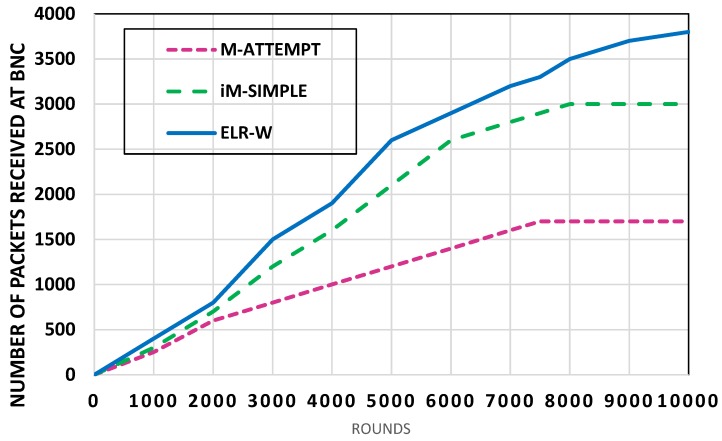
Analysis of network throughput.

The network throughput refers to the successful data transmitted to the destination. Figure 6 shows the analysis of throughput of the proposed ELR-W protocol in contrast to M-ATTEMPT and iM-SIMPLE. The Figure 6 indicates number of packets successfully received at BNC by M-ATTEMPT, iM-SIMPLE, and ELR-W are nearly 1700, 3000, and 3800 respectively. The ELR-W protocol achieved higher value of successful packets received due to the longer stability of individual BSNs. The BSNs died early in ATTEMPT and iM-SIMPLE which resulted in a lower number of packets received at BNC. Statistically, the throughput of ELR-W is 19% higher than iM-SIMPLE, and 102% higher than M-ATTEMP which is more than double. The M-ATTEMPT carried out low performance because of using thermal effect and mobility approach together. 

**Figure 7:**
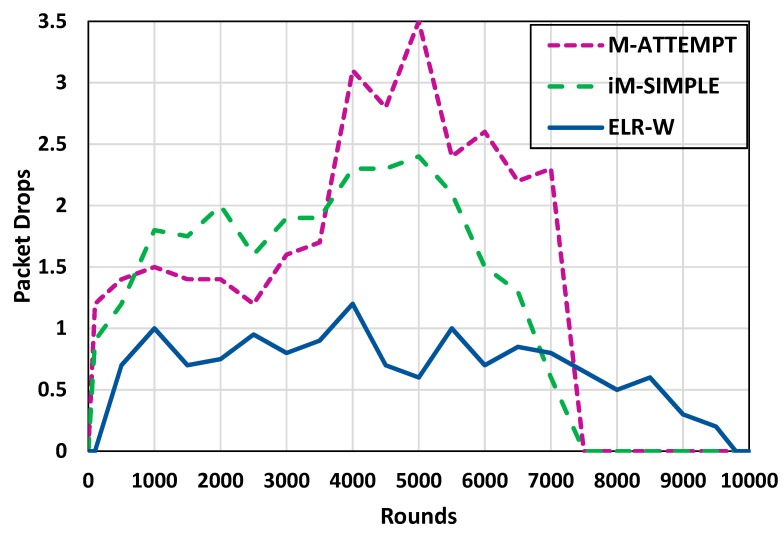
Analysis of packet drops.

In BSNs packet drops occur when data packets fail to reach the BNC. Packet drop assessment can be a critical parameter to measure the performance of a routing protocol. The throughput and packet drops are inversely proportional to each other. More throughput in the network results in a lower number of packet drops. Figure 7 presents packets drops analysis of ELR-W protocol in contrast to ATTEMPT and iM-SIMPLE. The analysis shows that the ELR-W drops a lower number of packets as compared to the competitive ones, which increases the reliability of ELR-W protocol.

**Figure 8:**
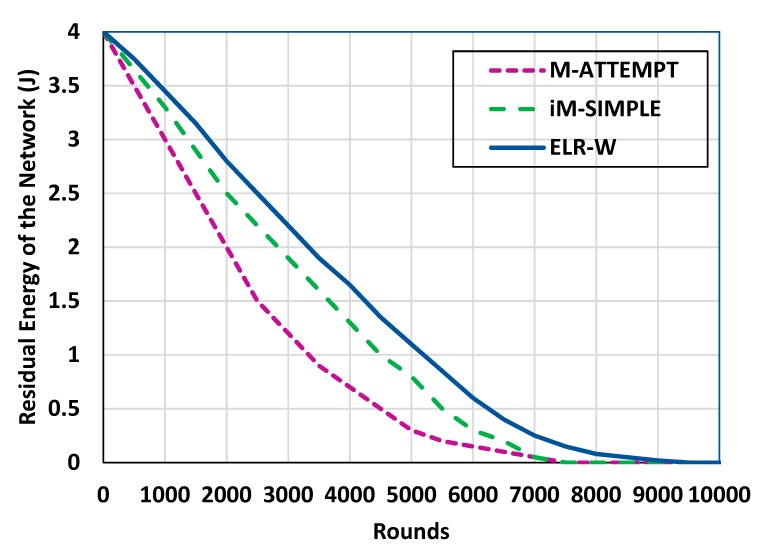
Analysis of energy consumption.

As a means to analyze the energy efficiency of the proposed ELR-W protocol, the energy consumption of the BSNs is observed in each round. Figure 8 shows the analysis of energy consumption of ELR-W against existing protocols which presents that the energy consumption of ELR-W is less than ATTEMPT and iM-SIMPLE. Moreover, it shows the residual energy is more stable than competitive protocols. Results show that the ELR-W consumes energy 14% and 45% less than iM-SIMPLE and M-ATTEMPT correspondingly. ELR-W achieved this because of using the effective criteria for the selection of next-hop in the network. The selection criteria are based on path cost expressed in Equation (4). The proposed path cost function supports the load balancing in the network which increases throughput along with a lower number of packet drops. As a result, there are fewer packet retransmission attempts in ELR-W protocol which reduces the overall energy consumption in the network. Table 3 and Table 4 show the performance of ELR-W in a nutshell as compared to the benchmark protocols.

It is noteworthy as shown in Table 4 that the ELR-W protocol outperforms 19% and 102% in terms of increased throughput, 30% and 34% in increased network lifetime and, 14% and 45% in reduced energy consumption as compared to the benchmark protocols iM-SIMPLE and M-ATTEMPT respectively. 

## 5. Conclusions

In this paper, we introduced a new routing protocol (ELR-W) for the purpose of achieving energy efficiency in WBANs. We introduced a novel path cost function contingent on residual energy, link efficiency, hop counts, and distance to the BNC for selection of the next-hop to transmit the data packets. We performed a series of experiments in NS-2 to analyze the performance of ELR-W for different criteria which included network lifetime, throughput, and energy consumption. The experimental results revealed less energy consumption and packet loss by ELR-W protocol which yielded high throughput and network lifetime in contrast to the state-of-the-art M-ATTEMPT and iM-SIMPLE protocols. Furthermore, this work can be further extended towards integration with Internet of Things (IoT) for monitoring of multiple WBANs in a hospital environment. 

## Figures and Tables

**Table 1:** HP header fields in ELR-W Protocol.

Symbol	Description
*SID*	Source node identifier
*NID*	Neighbor node identifier
*RE*	Residual energy
*LE*	Link efficiency between the nodes
*HC*	Number of hop-counts to the BNC
*d*	Distance from source node to BNC

**Table 2:** Simulation parameters.

Parameter	Value
Initial energy	0.5 Joule
Traffic type	CBR
Packet size	32 Bytes
Transmission power	10.5 mA
Reception power	18 mA
Transmitter electronics $\left(E_{Tx-elect}\right)$	16.7 nJ/bit
Receiver electronics $\left(E_{Rx-elect}\right)$	36.1 nJ/bit
Transmit amplifier $\left(E_{amp}\right)$	1.97 nJ/bit/mn
Supply voltage	1.9 V
Simulation time	100 s

**Table 3:** Analysis of: (A) network lifetime; (B) network throughput; (C) packet drops; (D) energy consumption.

	(A) Network Lifetime			(B) Network Throughput			(C) Packet Drops			(D) Energy Consumption		
Rounds	M-ATTEMPT	iM-SIMPLE	ELR-W	M-ATTEMPT	iM-SIMPLE	ELR-W	M-ATTEMPT	iM-SIMPLE	ELR-W	M-ATTEMPT	iM-SIMPLE	ELR-W
0	0	0	0	100	150	200	0	0	0	4	4	4
500	0	0	0	250	300	400	1.2	0.9	0	3.5	3.65	3.75
1000	3	0	0	450	550	650	1.4	1.2	0.7	3	3.3	3.45
1500	3	0	0	600	700	800	1.5	1.8	1	2.5	2.9	3.15
2000	3	0	0	700	950	1050	1.4	1.75	0.7	2	2.5	2.8
2500	3	0	0	800	1200	1500	1.4	2	0.75	1.5	2.2	2.5
3000	3	0	0	900	1400	1750	1.2	1.6	0.95	1.2	1.9	2.2
3500	3	0	0	1000	1600	1900	1.6	1.9	0.8	0.9	1.6	1.9
4000	3	1	0	1100	1900	2200	1.7	1.9	0.9	0.7	1.3	1.65
4500	3	1	0	1200	2100	2600	3.1	2.3	1.2	0.5	1	1.35
5000	3	3	0	1300	2300	2750	2.8	2.3	0.7	0.3	0.8	1.1
5500	3	4	0	1400	2600	2900	3.5	2.4	0.6	0.2	0.5	0.85
6000	3	5	1	1500	2700	3000	2.4	2.1	1	0.15	0.3	0.6
6500	3	6	1	1600	2800	3200	2.6	1.5	0.7	0.1	0.2	0.4
7000	4	7	1	1650	2900	3350	2.2	1.3	0.85	0.05	0.05	0.25
7500	5	8	3	1700	3000	3500	2.3	0.6	0.8	0	0	0.15
8000	8	8	4	1700	3000	3600	0	0	0.65	0	0	0.08
8500	8	8	5	1700	3000	3700	0	0	0.5	0	0	0.05
9000	8	8	6	1700	3000	3750	0	0	0.6	0	0	0.02
9500	8	8	6	1700	3000	3800	0	0	0.3	0	0	0
10,000	8	8	6	1700	3000	3800	0	0	0.3	0	0	0

**Table 4:** Performance of ELR-W against competitive protocols with increase↑or decrease↓trend.

Protocols	Performance of ELR-W against Benchmark Protocols		
	Throughput	Energy Consumption	Network Lifetime
iM-SIMPLE	19% ↑	14% ↓	30% ↑
M-ATTEMPT	102% ↑	45% ↓	34% ↑

## References

[1] Kaiwartya O., Abdullah A.H., Cao Y., Altameem A., Prasad M., Lin C.T., Liu X. (2016). Internet of vehicles: Motivation, layered architecture, network model, challenges, and future aspects. IEEE Access.

[2] Cao Y., Kaiwartya O., Zhuang Y., Ahmad N., Sun Y., Lloret J. (2018). A Decentralized Deadline-Driven Electric Vehicle Charging Recommendation. IEEE Syst. J..

[3] Kaiwartya O., Abdullah A.H., Cao Y., Raw R.S., Kumar S., Lobiyal D.K., Isnin I.F., Liu X., Shah R.R. (2016). T-MQM: Testbed-based multi-metric quality measurement of sensor deployment for precision agriculture—A case study. IEEE Sens. J..

[4] Qureshi K.N., Abdullah A.H., Kaiwartya O., Iqbal S., Butt R.A., Bashir F. (2017). A Dynamic Congestion Control Scheme for safety applications in vehicular ad hoc networks. Comput. Electr. Eng..

[5] Kasana R., Kumar S., Kaiwartya O., Kharel R., Lloret J., Aslam N., Wang T. (2018). Fuzzy based Channel Selection for Location Oriented Services in Multichannel VCPS Environments. IEEE Internet Things J..

[6] Keehan S.P., Cuckler G.A., Sisko A.M., Madison A.J., Smith S.D., Stone D.A., Poisal J.A., Wolfe C.J., Lizonitz J.M. (2015). National Health Expenditure Projections, 2014−24: Spending Growth Faster Than Recent Trends. Health Aff..

[7] World Health Organization (2016). Global Report on Diabetes.

[8] Anwar M., Anwar A.H., Abdullah A.H., Abdullah K.N., Qureshi K.N., Majid A.H. (2017). Wireless Body Area Networks for Healthcare Applications: An Overview. Telkomnika.

[9] Masud F., Masud A.H., Abdullah A.H., Abdul-Salaam G., Ullah F. (2017). Traffic adaptive MAC protocols in wireless body area networks. Wirel. Commun. Mob. Comput..

[10] Ullah F., Ullah A.H., Abdullah A.H., Kaiwartya O., Cao Y. (2017). TraPy-MAC: Traffic Priority Aware Medium Access Control Protocol for Wireless Body Area Network. J. Med. Syst..

[11] Ko J.G., Lu C., Lu M.B., Srivastava M.B., Stankovic J.A., Terzis A., Welsh M. (2010). Wireless sensor networks for healthcare. Proc. IEEE.

[12] Wang H., Md Shaad M., Fang H., Wang C. (2016). Wireless Health.

[13] Cavallari R., Martelli F., Rosini R., Buratti C., Verdone R. (2014). A Survey on Wireless Body Area Networks: Technologies and Design Challenges. IEEE Commun. Surv. Tutor..

[14] Lai X., Liu Q., Wei X., Wang W., Zhou G., Han G. (2013). A survey of body sensor networks. Sensors.

[15] Ullah F., Ullah A.H., Abdullah A.H., Kaiwartya O., Lloret J., Arshad M.M. (2017). EETP-MAC: Energy efficient traffic prioritization for medium access control in wireless body area networks. Telecommun. Syst..

[16] Ishtaique ul Huque T., Munasinghe K.S., Abolhasan M., Jamalipour A. EAR-BAN: Energy Efficient Adaptive Routing in Wireless Body Area Networks. Proceedings of the 7th International Conference on Signal Processing and Communication Systems (ICSPCS).

[17] Lee S., Annavaram M. Wireless Body Area Networks: Where does energy go?. Proceedings of the 2012 International Symposium on Workload Characterization.

[18] Ahmed G., Jianhua Z., Fareed M.M.S. (2017). PERA: Priority-Based Energy-Efficient Routing Algorithm for WBANs. Wirel. Pers. Commun..

[19] Kaur N., Singh S. (2017). Optimized cost effective and energy efficient routing protocol for wireless body area networks. Ad Hoc Netw..

[20] Ruzzelli A.G., Jurdak R., Jurdak G.M.P., O’Hare G.M.P., Van Der Stok P. Energy-Efficient Multi-hop Medical Sensor Networking. Proceedings of the 1st ACM SIGMOBILE International Workshop on Systems and Networking Support for Healthcare and Assisted Living Environments–HealthNet ′07.

[21] Yuce M.R. (2010). Implementation of wireless body area networks for healthcare systems. Sens. Actuators A-Phys..

[22] Qureshi K.N., Qureshi A.H., Abdullah A.H., Bashir F., Iqbal S., Awan K.M. (2018). Cluster-based data dissemination, cluster head formation under sparse, and dense traffic conditions for vehicular ad hoc networks. Int. J. Commun. Syst..

[23] Javaid N., Abbas Z., Abbas M.S., Fareed M.S., Fareed Z.A., Khan Z.A., Alrajeh N. (2013). M-ATTEMPT: A new energy-efficient routing protocol for wireless body area sensor networks. Procedia Comput. Sci..

[24] Effatparvar M., Dehghan M., Rahmani A.M. (2016). A comprehensive survey of energy-aware routing protocols in wireless body area sensor networks. J. Med. Syst..

[25] Maskooki A., Maskooki C.B., Soh C.B., Gunawan E., Low K.S. Opportunistic routing for body area network. Proceedings of the 2011 IEEE Consumer Communications and Networking Conference (CCNC).

[26] Javaid N., Ahmad A., Nadeem Q., Imran M., Haider N. (2015). IM-SIMPLE: IMproved Stable Increased-throughput Multi-hop Link efficient Routing Protocol for Wireless Body Area Networks. Comput. Hum. Behav..

[27] Liang L., Ge Y., Feng G., Ni W., Wai A.A.P. (2014). A low overhead tree-based energy-efficient routing scheme for multi-hop wireless body area networks. Comput. Netw..

[28] Ahmed S., Javaid N., Yousaf S., Ahmad A., Sandhu M.M., Imran M., Khan Z.A., Alrajeh N. (2015). Co-LAEEBA: Cooperative link aware and energy efficient protocol for wireless body area networks. Comput. Hum. Behav..

[29] Kim B.-S., Kim K., Kim K.-I. (2017). A Survey on Mobility Support in Wireless Body Area Networks. Sensors.

[30] Nadeem Q., Javaid N., Javaid S.N., Mohammad S.N., Mohammad M.Y., Khan M.Y., Sarfraz S., Gull M. SIMPLE: Stable increased-throughput multi-hop protocol for link efficiency in Wireless Body Area Networks. Proceedings of the IEEE 8th International Conference on Broadband and Wireless Computing, Communication and Applications (BWCCA′13).

[31] Sandhu M.M., Javaid N., Imran M., Guizani M., Khan Z.A., Qasim U. (2015). BEC: A Novel Routing Protocol for Balanced Energy Consumption in Wireless Body Area Networks. IWCMC.

[32] Yessad N., Omar M., Tari A., Bouabdallah A. (2017). QoS-based routing in Wireless Body Area Networks: A survey and taxonomy. Computing.

[33] Adhikary S., Choudhury S., Chattopadhyay S. A new routing protocol for WBAN to enhance energy consumption and network lifetime. Proceedings of the 17th International Conference on Distributed Computing and Networking.

[34] Ha I. (2016). Even energy consumption and backside routing: An improved routing protocol for effective data transmission in wireless body area networks. Int. J. Distrib. Sens. Netw..

[35] Ayatollahitafti V., Ayatollahitafti M.A., Ngadi M.A., Ngadi J.B.M., Sharif J.B.M., Abdullahi M. (2016). An efficient next hop selection algorithm for multi-hop body area networks. PLoS ONE.

[36] Ullah Z., Ahmed I., Razzaq K., Razzaq M.K., Naseer M.K., Ahmed N. (2017). DSCB: Dual sink approach using clustering in body area network. Peer-to-Peer Netw. Appl..

[37] Baccour N., Puccinelli D., Voigt T., Koubaa A., Noda C., Fotouhi H., Alves M., Youssef H., Zuniga M.A., Boano C.A. (2013). Overview of Link Quality Estimation. Radio Link Quality Estimation in Low-Power Wireless Networks.

[38] Dijkstra E.W. (1959). A Note on two Problems in Connexion with Graphs. Numer. Math..

[39] Heinzelman W.R., Chandrakasan A., Balakrishnan H. Energy-efficient communication protocol for wireless microsensor networks. Proceedings of the 33rd Annual Hawaii International Conference on System Sciences.

[40] Chávez-Santiago R., Garcia-Pardo C., Fornes-Leal A., Vallés-Lluch A., Vermeeren G., Joseph W., Balasingham I., Cardona N. (2015). Experimental Path Loss Models for In-Body Communications within 2.36–32.5 GHz. IEEE J. Biomed. Health Inform..

[41] Maity S., Mojabe K., Sen S. (2018). Characterization of Human Body Forward Path Loss and Variability Effects in Voltage-Mode HBC. IEEE Microw. Wirel. Compon. Lett..

[42] Kurup D., Vermeeren G., Tanghe E., Joseph W., Martens L. (2015). In-to-Out Body Antenna-Independent Path Loss Model for Multilayered Tissues and Heterogeneous Medium. Sensors.

[43] Hausman S., Januszkiewicz Ł. (2014). Impact of Indoor Environment on Path Loss in Body Area Networks. Sensors.

[44] Yousaf S., Javaid N., Qasim U., Alrajeh N., Khan Z., Ahmed M. (2016). Towards Reliable and Energy-Efficient Incremental Cooperative Communication for Wireless Body Area Networks. Sensors.

[45] Reusens E., Joseph W., Latré B., Braem B., Vermeeren G., Tanghe E., Martens L., Moerman I., Blondia C. (2009). Characterization of on-body communication channel and energy efficient topology design for wireless body area networks. IEEE Trans. Inf. Technol. Biomed..

[46] Rappaport T.S. (1996). Wireless Communications: Principles and Practice.

[47] Semiconductor N. (2007). Single Chip 2.4 GHz Transceiver Product Specification.

